# GIDEON: a computer program for diagnosis, simulation, and informatics in the fields of geographic medicine and emerging diseases.

**DOI:** 10.3201/eid0707.017729

**Published:** 2001

**Authors:** S A Berger

**Affiliations:** Tel Aviv Medical Center, Tel Aviv, Israel. mberger@post.tau.ac.il


The posteradication phase (high containment) begins 1 year after identification of the world's last case. At that time, laboratories holding wild poliovirus stocks and potentially infectious materials must either place all materials under appropriate biosafety conditions, transfer important virus isolates to WHO interim repositories, or render all wild poliovirus materials noninfectious. Documentation of containment compliance by all regions is required for global certification of poliovirus eradication. For countries that intend to stop all poliovirus vaccination, work with materials that could cause infection with wild poliovirus must be conducted under BSL 4 containment. High containment (BSL 3/polio) will be required for work with vaccine-derived viruses.

High-level political involvement and multi-sector commitments, including departments of health, defense, education, environment, and private industry are essential to achieving and maintaining global containment of wild poliovirus.

### References

1. Mulders MN, Reimerink JHJ, Koopmans MPG, van Loon AM, van der Avoort HGAM. Genetic analysis of wild type poliovirus importation into The Netherlands (1979-1995). J Infect Dis 1997;176:617-24.
2. World Health Organization. Global action plan for laboratory containment of wild polioviruses. Geneva, Switzerland: World Health Organization; 1999.

### GIDEON: A Computer Program for Diagnosis, Simulation, and Informatics in the Fields of Geographic Medicine and Emerging Diseases

**Stephen A. Berger**
Tel Aviv Medical Center, Tel Aviv, Israel

Over 300 infectious diseases occur and are challenged by over 250 drugs and vaccines. Fifteen hundred species of pathogenic bacteria, viruses, parasites, and fungi have been described, and printed media can no longer keep up with the dynamics of diseases, outbreaks, and epidemics in "real time." Although electronic media have given us unlimited information access, the search for meaningful data is confusing and time-consuming. Global Infectious Diseases and Epidemiology Network (GIDEON) is a computer software program that was developed for disease simulation and informatics in the fields of geographic and travel medicine.

GIDEON is currently used in 1,500 sites in 45 countries: health ministries, military installations, travel clinics, libraries and student teaching modules, clinical departments, laboratories, and missionary agencies. The program consists of four components. The first generates a Bayesian ranked differential diagnosis based on signs, symptoms, laboratory tests, country of origin, and incubation period and can be used for diagnostic support and simulation of all infectious diseases in all countries. In a blind trial conducted on 495 patients, the correct diagnosis was included in the differential diagnosis list in 94.7% of cases (sensitivity) and displayed as the first disease in the list in 75% (specificity).

The second component presents the epidemiology of individual diseases, including their global effects and status in each of 205 countries and regions. All past and current outbreaks are described in detail, and a web-based version under development will allow for daily updating online. The user may also access a list of diseases related to any agent, vector, vehicle, reservoir or country or any combination of all five (i.e., a list of all mosquito-borne viruses of Brazil which have an avian reservoir).

The third module is an interactive encyclopedia which includes information on the pharmacology, use, testing standards, and global trade names of all antiinfective drugs and vaccines.

The fourth module is designed to identify all species of bacteria, mycobacteria, and yeasts. The database includes 50 to 100 additional taxa that may not appear in standard texts and laboratory databases for several months. Other options allow the user to add data relevant to his own institution, electronic patient charts, material from the Internet, important telephone numbers, drug prices, antimicrobial resistance patterns, and other information. This form of custom data input is particularly useful when running GIDEON on institutional networks because software administrators can use it to disseminate and file information relevant to their own institution for use by all computers on their network. The data in GIDEON are derived from all peer-reviewed journals in the fields of infectious diseases, pediatrics, internal medicine, tropical medicine, travel medicine, antimicrobial pharmacology, and clinical microbiology; a monthly electronic literature search based on all relevant terms in GIDEON (e.g., diseases, drugs, etc.) all available health ministry reports (both printed and electronic); standard texts; and abstracts of major meetings. Further details regarding the program are available at http://www.cyinfo.com.

Address for correspondence: Stephen A. Berger, 6 Weitzman Street, Tel Aviv 64239, Israel; fax: 972-3-6132892; e-mail: mberger@post.tau.ac.il
